# Excellent 10-year patient-reported outcomes and survival in a single-radius, cruciate-retaining total knee arthroplasty

**DOI:** 10.1007/s00167-018-5179-9

**Published:** 2018-10-01

**Authors:** Chloe E. H. Scott, Katrina R. Bell, Richard T. Ng, Deborah J. MacDonald, James T. Patton, Richard Burnett

**Affiliations:** 1 Department of Orthopaedics, Royal Infirmary of Edinburgh, 51 Little France Crescent, Old Dalkeith Road, Edinburgh, EH16 4SA UK; 2 Department of Orthopaedics, University of Calgary, 401 9th Ave SW, Suite 335, Calgary, AB T2P 3C5 Canada

**Keywords:** Total knee arthroplasty, Single-radius knee, Long-term survival, Patient-reported outcomes

## Abstract

**Purpose:**

Over 2 million Triathlon single-radius total knee arthroplasties (TKAs) have been implanted worldwide. This study reports the 10-year survival and patient-reported outcome of the Triathlon TKA in a single independent centre.

**Methods:**

From 2006 to 2007, 462 consecutive cruciate-retaining Triathlon TKAs were implanted in 426 patients (median age 69 (21–89), 289 (62.5%) female). Patellae were not routinely resurfaced. Patient-reported outcome measures (SF-12, Oxford Knee Scores (OKS), satisfaction) were assessed preoperatively and at 1, 5 and 10 years when radiographs were reviewed. Forgotten Joint Scores (FJS) were collected at 10 years. Kaplan–Meier survival analysis was performed.

**Results:**

At 10–11.6 years, 123 patients (128 TKAs) had died and 8 TKAs were lost to follow-up. There were four aseptic failures (two cases of tibial loosening, two cases of instability) and four septic failures requiring revision. Symptomatic aseptic radiographic loosening was present in three further cases at 11 years. Four (1%) patellae were secondarily resurfaced. OKS score improved by 17.7 ± 9.7 points at 1 year (*p* < 0.001), and was maintained at 34.7 ± 9.6 at 10 years with FJS 48.5 ± 31.4. Patient satisfaction was 88% at each timepoint. Ten-year survival was 97.9% (95% confidence interval 96.5–99.3) for revision for any reason, 98.9% (97.7–100) for mechanical failure, and 98.6% (97.4–99.8) for aseptic loosening (symptomatic radiographic or revised).

**Conclusion:**

The Triathlon TKA continues to show excellent longer-term results with high implant survivorship, low rates of aseptic failure, consistently maintained PROMs and excellent patient satisfaction rates of 88% at 10 years.

**Level of evidence:**

II, Prospective cohort study.

## Introduction

The number of total knee arthroplasties (TKAs) performed annually in the United Kingdom continues to rise with projections suggesting this is to continue at least to 2035 in the United Kingdom [12] and internationally [27, 31]. Though TKA is a cost-effective treatment for end-stage degenerative joint disease [29], patient dissatisfaction rates of 15–20% are consistently reported [3, 36]. Dissatisfaction is highest in younger patients (< 55 years) [35] who at present are both the fastest growing utilisers of TKA and have the highest revision rates [31, 37]. There are therefore both patient-centred and population-centred drivers to improve functional outcome in TKA. Though predictors of outcomes are complex and multifactorial, implant design is potentially significant.

The design of condylar resurfacing TKA femoral components has followed the theory of a dynamic flexion–extension axis (FEA) following a J-shaped curve throughout a range of motion [18] since 1976 [28]. More recent cadaveric [6, 25] and three-dimensional imaging studies [16, 26] have suggested an alternative flexion–extension axis at the knee, common throughout a range of motion. This common FEA approximates to the surgical epicondylar axis [1, 16] and has a consistent relationship with both the patellofemoral joint axis [11] and the longitudinal rotational axis of the tibia [26]. It is a consistent feature in both varus and valgus knees [26]. This modern kinematic theory has been adopted in single-radius implant designs since 1996 and has more recently been combined with deep flexion adaptations in the Triathlon TKA. Though over 2 million Triathlon TKAs have now been implanted worldwide, no independent reports of mid- to long-term survival or outcomes of this design are yet published.

A single common flexion–extension axis at the knee conveys several theoretical biomechanical advantages. Ligament isometry throughout the range of movement facilitates more conforming polyethylene thus reducing contact stresses in addition to reducing mid-flexion instability. The more posterior location of the common FEA compared to J-shaped axes, lengthens the quadriceps moment arm providing a theoretical mechanical advantage to knee extension power [20] with a reduction in joint reaction force at the patellofemoral joint. Additional features of the Triathlon TKA include shorter posterior condyles with consistent posterior condylar offset between sizes to encourage deep flexion.

In 2016 the Triathlon TKA was the most common TKA prosthesis implanted in Australia and the third most implanted in the United Kingdom. Despite this, to date there have been no independent studies of mid- to long-term functional outcomes, radiographic reviews or details of complications other than revision which is proved in registry data. Ten-year survival is 96.1 (95% confidence interval (95% CI) 95.5–96.6) for the cruciate retaining Triathlon in the Australian registry 96.8% (95% CI 96.3–97.2) in the National Joint Registry of England and Wales. Good survival and functional outcomes have previously been reported at 5 years [34]. The aim of this study was to report the 10-year survival of the Triathlon TKA from a single independent centre. Secondary aims included radiographic assessment and patient-reported outcome measures (PROMs) of function and pain to determine the longitudinal outcomes over 10 years.

## Materials and methods

From 2006 to 2007, data were recorded for consecutive patients undergoing Triathlon TKAs (Stryker Orthopaedics, Mahwah, NJ, USA) performed or supervised by seven consultant surgeons at a single large orthopaedic teaching hospital. Cemented, cruciate-retaining TKAs with standard tibial baseplates were performed in all cases via a medial para-patella approach using a tourniquet. Simplex bone cement (Stryker Orthopaedics, Mahwah, NJ, USA) was used both with (*n* = 349) and without (*n* = 113) gentamicin. The patella was not routinely resurfaced. The patella was resurfaced primarily in 24 patients (5.2%) at the surgeons’ discretion. Antibiotic prophylaxis was with three doses cefuroxime. Standard primary implants were used in all but one patient where a medial tibial plateau fracture non-union required medial augmentation and tibial stem. A CR implant was used in this case. Four TKAs were navigated (surgeon discretion). Of the 36 patients who underwent bilateral TKAs, 9 patients had 18 TKAs performed as bilateral simultaneous procedures. All patients followed standardised post-operative rehabilitation with mobilisation from day 1 and discharge home when independently mobile with two sticks.

### PROMs

Prior to surgery, a postal questionnaire including the Short-form (SF-12) general health questionnaire [15] and the knee-specific Oxford Knee Score [13] (OKS) was sent to all patients. This was collected in a pre-assessment clinic 3 weeks prior to surgery. The SF-12 is a validated generic health questionnaire with physical and mental health components. Following TKA the minimal clinically important difference (MCID) in the physical component score is 4.5 for pain and 4.8 for function [8]. The OKS is a validated knee-specific outcome measure of 12 questions with 5 possible answers giving a score from 0 to 48 [13] and a MCID of 5 [8]. Higher scores represent better function. Completed questionnaires were collected at a nurse-led pre-assessment clinic.

**Fig. 1:**
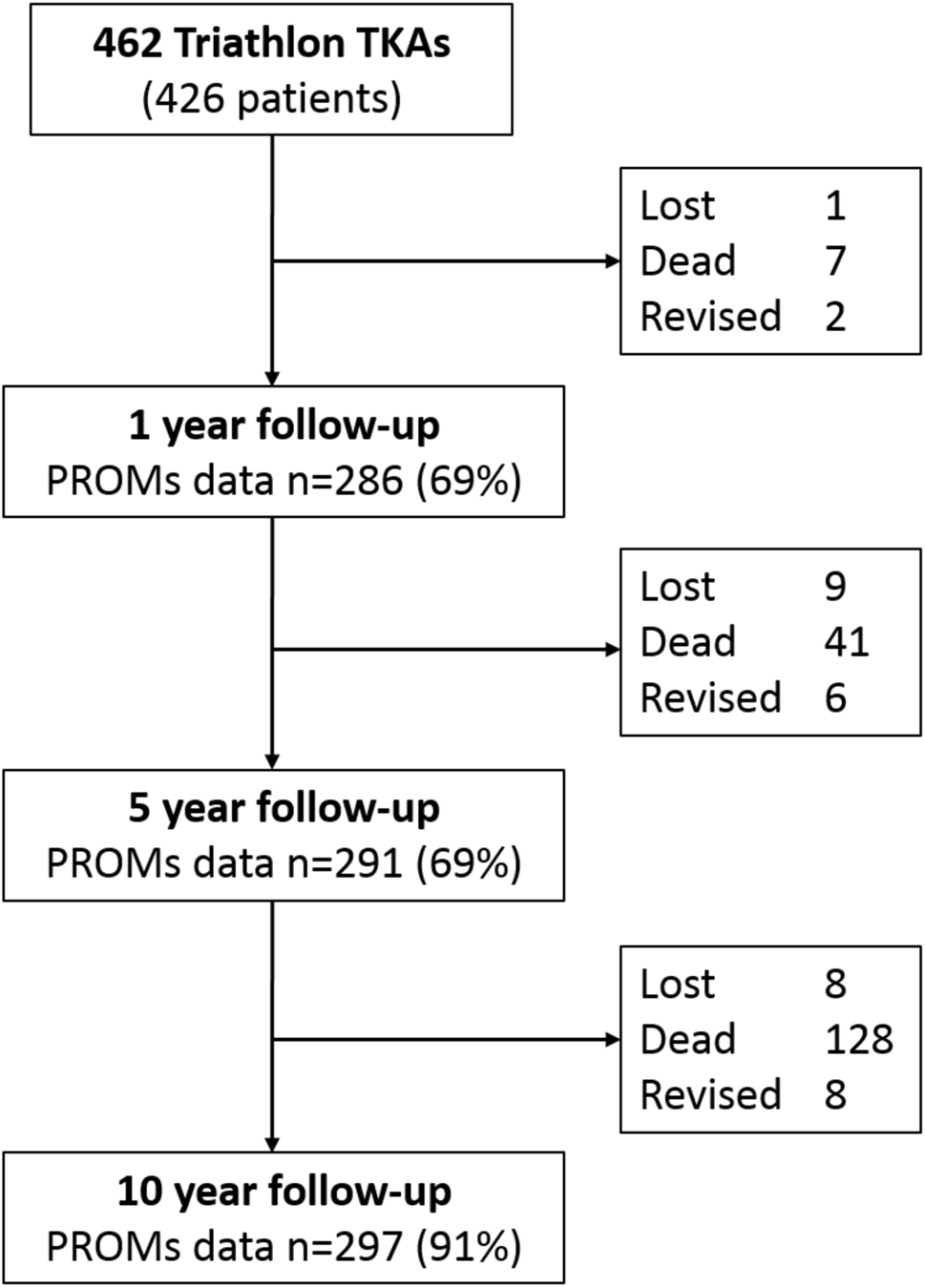
Study cohort with follow-up at each timepoint. All figures in the boxes on the right are cumulative

Post-operative questionnaires were sent to patients at 0.5, 1, 5 and 10 years. Response rates are detailed in Fig. 1. In addition to SF-12 and Oxford Knee Scores, questionnaires at and beyond 1 year included measures of patient satisfaction. Patients were asked, ‘How satisfied are you with your operated knee?’ with options ‘very satisfied’, ‘satisfied’, ‘unsure’ or ‘dissatisfied’ [36]. At 5 and 10 years patients were asked if they had undergone any reoperations and the nature of these. At 10 years the Forgotten Joint Score (FJS) was also collected. The FJS is a validated PROM outcome measure following knee arthroplasty designed to minimise ceiling effects in high-functioning individuals following knee arthroplasty [21]. Collection of data was independent of the routine clinical care of the patient. Patients who did not respond to the 5- and 10-year questionnaires were contacted by telephone and completed the questionnaire verbally.

### Survival

Medical and operation notes were examined for all patients. The patients’ demographics, indication, consultant in charge of care, date of surgery, and side were recorded. All intraoperative, early or late complications and their nature were recorded. Deep infection was defined as clinical infection with an identified organism; cellulitis was defined as superficial erythema with no apparent joint involvement which resolved with oral antibiotics; wound dehiscence was secondary gaping of part of the surgical wound requiring surgical intervention; and prolonged wound leakage was leakage which resolved in the absence of signs suggesting deep infection (pain, decreased range of motion, sepsis, organism on aspiration). In those patients who had undergone revision surgery, the mode of implant failure confirmed at revision was noted. Any other reoperation was also noted. Deceased patients were identified and date of death confirmed.

### Radiographic review

Short-leg weight-bearing radiographs taken at most recent orthopaedic review were examined by two independent reviewers (KRB and RTN) who had no clinical contact with the patients. Coronal and sagittal implant alignment was measured to one decimal place and has been reported previously [34]. Periprosthetic radiolucencies were reported in femoral and tibial regions as per the Knee Society Score [17]. If radiolucencies were present, all radiographs pertaining to that TKA were independently examined by both reviewers to assess progression. Where > 2 radiolucencies or any ballooning osteolysis was present, radiographs were additionally reviewed by a senior author (CEHS). The coronal and sagittal plane implant alignment have been reported previously [34].

Ethical approval was obtained for this prospective cohort study (Scotland (A) Research Ethics Committee 16/SS/0026). This study was completed without external funding.

### Statistical analysis

Data were analysed using SPSS version 21.0. Interobserver agreement of radiolucencies was tested using the Kappa statistic. Repeated measures ANOVA was used to examine changes in parametric variables over the 10-year study period. A *p* value of < 0.05 was considered to be statistically significant. Post hoc analysis of longitudinal PROMs was performed using paired *t* tests for parametric variables and Wilcoxon signed rank for non-parametric variables with significance set at *p* < 0.01 incorporating a Bonferroni correction to adjust for multiple testing at 5 points over 10 years. Survival analysis was undertaken with life-tables and Kaplan–Meier analysis. The endpoints used were revision for any reason, mechanical failure (aseptic loosening and instability), aseptic failure (including symptomatic radiographic and revision for aseptic loosening), and a worst-case-scenario analysis assuming that all TKAs lost to follow-up had failed.

## Results

In the study period, 462 consecutive cruciate retaining Triathlon TKAs were implanted in 426 patients. Median age was 69 years (range 21–89) and 289 (62.5%) were female. The indication for surgery was primary osteoarthritis in 406/462 (87.9%).

During the study (Fig. 1), 123 patients (128 TKAs (27.7%)) had died with their implant intact. At minimum follow-up of 10 years (mean 10.8, SD 0.38) PROMs were obtained for 297 of 326 remaining TKAs (91%). Eight patients (8 TKAs (1.7%)) were uncontactable and were considered lost. One patient uncontactable at 5 years was located at 10 years. Other non-responders (*n* = 21) included 11 (2.4%) patients with dementia and 10 (2.2%) who were contacted, but refused to complete questionnaires though had intact TKAs.

### Complications and survival analysis

**Fig. 2:**
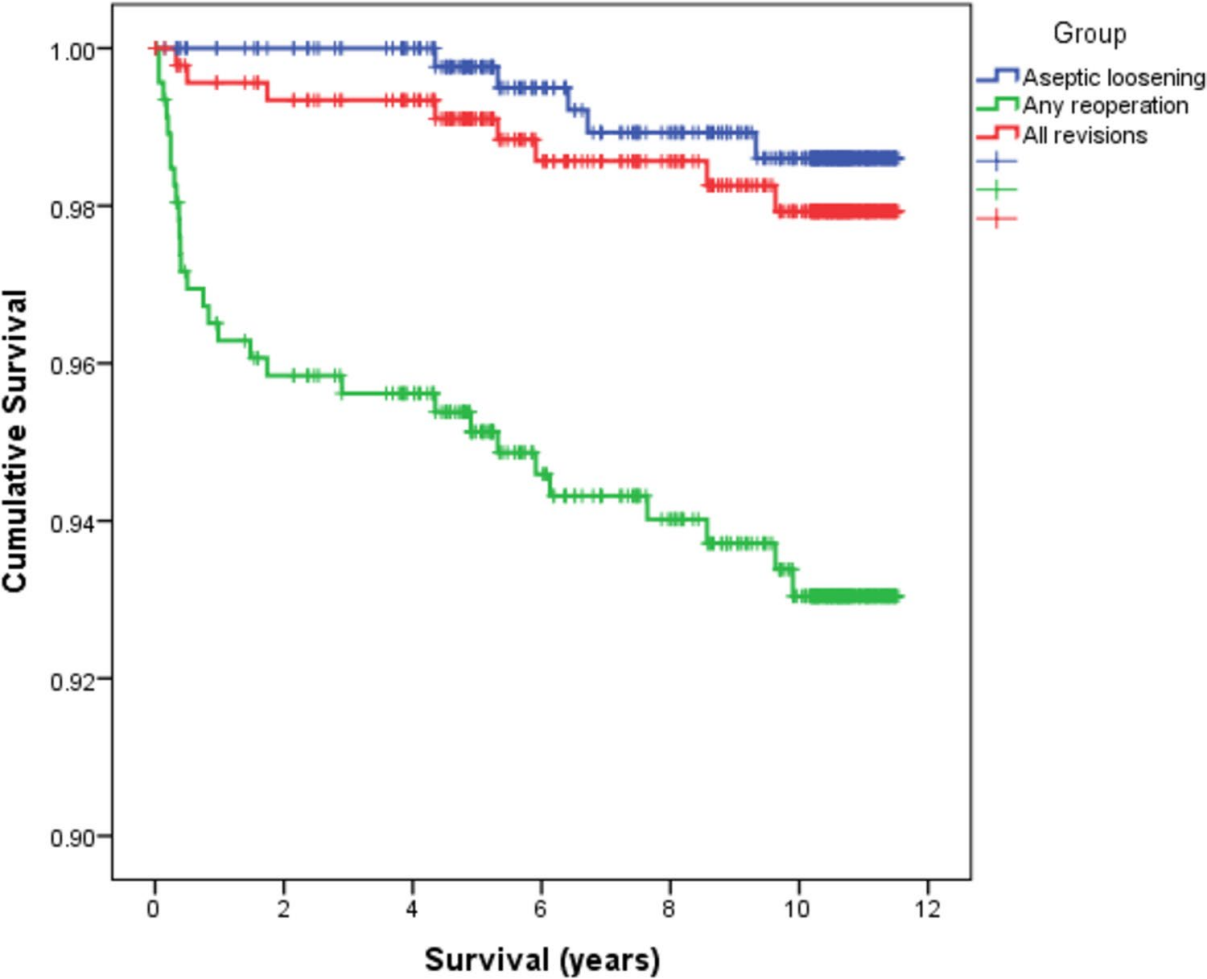
Kaplan–Meier survival analyses for the endpoints aseptic loosening (revised or symptomatic radiographic), all revisions and all reoperations (including revision)

Early (< 6 weeks) and late complications (> 6 weeks) and reoperations are detailed in Table 1. Deep infections were managed with debridement and implant retention in three cases (two with long-term antibiotic suppression) and four with revision. There were four other revisions during the study period undertaken for mechanical reasons, including two cases of tibial aseptic loosening. Radiographic review identified three additional cases of symptomatic radiographic tibial loosening. The life table for all revisions is given in Table 2. Individual failures are detailed in Table 3. Ten-year Kaplan–Meier survival analyses are shown in Table 4 and Fig. 2.

**Table 1:** Complications

Complication	Number (%)
Early	
Prolonged wound leak	19 (4.1)
Wound dehiscence	1 (0.2)
Cellulitis	2 (0.4)
Deep infection	1 (0.2)
VTE	9 (1.9)
Myocardial infarction	3 (0.6)
Late	
Infection	7 (1.5)
Instability	6 (1.3)
Tibial loosening	2 (0.4)
Periprosthetic fracture	3 (0.6)
Anterior knee pain	19 (4.1)
Unexplained pain	10 (2.2)
Stiffness	2 (0.4)
Reoperations (not revision)	
All	21 (4.5)
Secondary wound closure	1 (0.2)
Debridement and implant retention	3 (0.6)
Manipulation under anaesthesia	10 (2.2)
Arthrolysis	1 (0.2)
Secondary resurfacing	4 (0.9)
Open reduction and internal fixation	2 (0.4)

*VTE* venous thromboembolism

**Table 2:** Life table for total knee arthroplasty failures requiring revision

Interval (years)	Number	Failures	Lost	Withdrawn	At risk	Failure rate (%)	Cumulative survival (%)	95% CI	
								Lower limit	Upper limit
0–1	462	2	1	7	458	0.4	99.6	99.0	100
1–2	452	1	0	4	450	0.2	99.4	99.0	99.8
2–3	447	0	1	5	444	0	99.4	99.0	99.8
3–4	441	0	0	5	438	0	99.4	99.0	99.8
4–5	436	1	2	15	427	0.2	99.2	98.8	99.6
5–6	418	2	0	12	412	0.5	98.7	98.1	99.3
6–7	404	0	2	14	396	0	98.7	98.1	99.3
7–8	388	0	1	12	381	0	98.7	98.1	99.3
8–9	375	1	1	21	364	0.3	98.4	97.8	99.0
9–10	352	1	0	23	340	0.3	98.1	97.5	98.7
10–11	328	0	0	213	221	0	98.1	97.5	98.7
> 11	115	0	0	115	57	0	98.1	97.5	98.7

*CI* confidence interval

**Table 3:** Details of individual total knee arthroplasty failures

Age	Sex	Indication	Survival (years)	Mode of failure	Management
74	M	OA	0.25	Infection	Two-stage revision
82	M	OA	0.33	Infection	DAIR and long-term suppression
57	M	OA	0.5	Infection	Two-stage revision
62	M	OA	1.67	Infection	Two-stage revision
71	F	#NU	3.75	Infection	DAIR and long-term suppression
61	F	OA	4.3	Aseptic loosening tibia	Revision
55	F	RA	5.25	Aseptic loosening tibia	Revision
52	F	RA	5.8	Instability	Revision
74	F	OA	8.6	Infection	Two-stage revision
45	F	OA	9.6	Instability	Revised

*M* male, *F* female, *OA* osteoarthritis, *RA* rheumatoid arthritis, # fracture, *NU* non-union, *DAIR* debridement and implant retention

**Table 4:** Ten-year Kaplan–Meier survival functions for different end points

End point	*n*	Survival % (95% CI)
Any revision	8	97.9 (96.5–99.3)
Mechanical failure (aseptic loosening or instability)	4	98.9 (97.7–100)
Aseptic loosening (revised or symptomatic radiographic)	5	98.6 (97.4–99.8)
Any reoperation (including revisions)	29	93.0 (90.5–95.6)
Worst-case scenario (revised or lost)	23	94.4 (92.1–96.8)

### Radiographic outcome

Mean medial proximal tibial angle was 89.8° ± 1.9°; posterior tibial slope 2.7° ± 2.3°; femoral valgus angle 2.0° ± 1.9°; and femoral component flexion 15.7° ± 4.1° as previously published [34]. Radiographic review at mean 8.22 ± 1.94 years of 266 TKAs identified: 1 case of definite symptomatic radiographic failure (tibial loosening with osteolysis); 9 concerning for loosening (2 symptomatic—3 tibial only, 5 femoral only, 1 both); 11 with > 2 zones [17] of lucency and 105 with ≤ 2 zones [17] of lucency consistent with cementation defects (zones 1 and 4 tibia or femur).

### Patient-reported outcome measures

SF-12 physical component scores (*p* < 0.001) changed significantly over the study period (Table 5) with the greatest change in the first 6 months: 30.2 ± 7.15 to 41.5 ± 10.0 (*p* < 0.001). Mental component scores did not change significantly over the study period (*p* = 0.014).

**Table 5:** Absolute PROMs at each timepoint with improvements in OKS for individuals

PROM	Timepoint	Median	Mean (95% CI)	*p* value
PCS	Preop	29.0	30.5 (29.3 to 31.8)	< 0.001^^^
	0.5 year	42.1	41.5 (39.8 to 43.1)	
	1 year	43.4	43.4 (41.5 to 45.2)	
	5 years	39.3	41.7 (39.7 to 43.8)	
	10 years	39.6	39.2 (37.2 to 41.2)	
MCS	Preop	53.6	51.3 (49.4 to 53.2)	0.014^^^
	0.5 year	55.4	52.3 (50.6 to 54.0)	
	1 year	55.9	52.6 (50.9 to 54.4)	
	5 years	54.4	51.7 (49.9 to 53.5)	
	10 years	50.9	48.6 (46.7 to 50.5)	
OKS	Preop	18	18.8 (17.6 to 19.9)	< 0.001^^^
	0.5 year	37	34.3 (32.6 to 36.1)	
	1 year	39	36.3 (34.6 to 38.0)	
	5 years	41	37.3 (35.5 to 39.0)	
	10 years	38	34.7 (32.9 to 36.5)	
OKS improvement	Preop to 1 year	18	17.7 (16.1 to 19.2)	< 0.001*
	1–5 years	1	1.2 (− 0.1 to 2.4)	n.s.*
	5–10 years	− 1.5	− 3.0 (− 4.3 to − 1.7)	< 0.001*

*p* values (^^^two-way ANOVA, *paired *t* tests) reflect changes over time between values

**Fig. 3:**
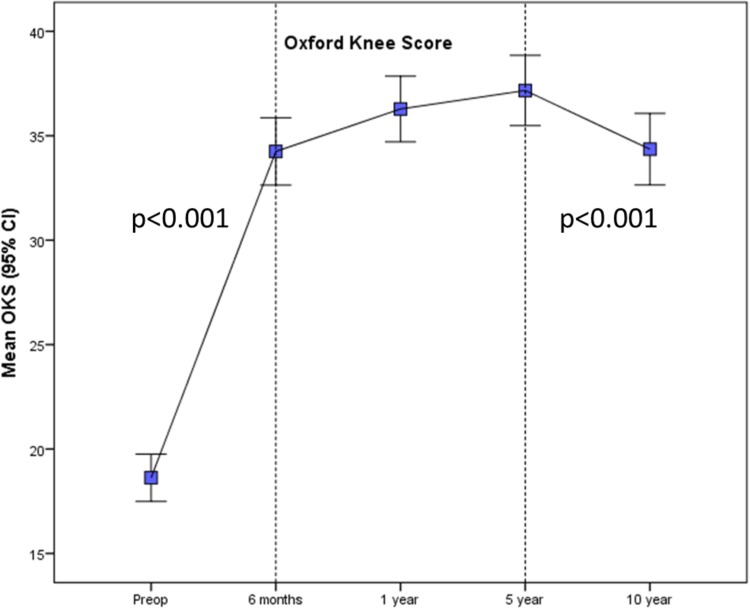
Longitudinal Oxford Knee Scores (OKS) over the 10-year study period. Significant improvement occurred over the first year with a small but significant decline from 5 to 10 years

**Fig. 4:**
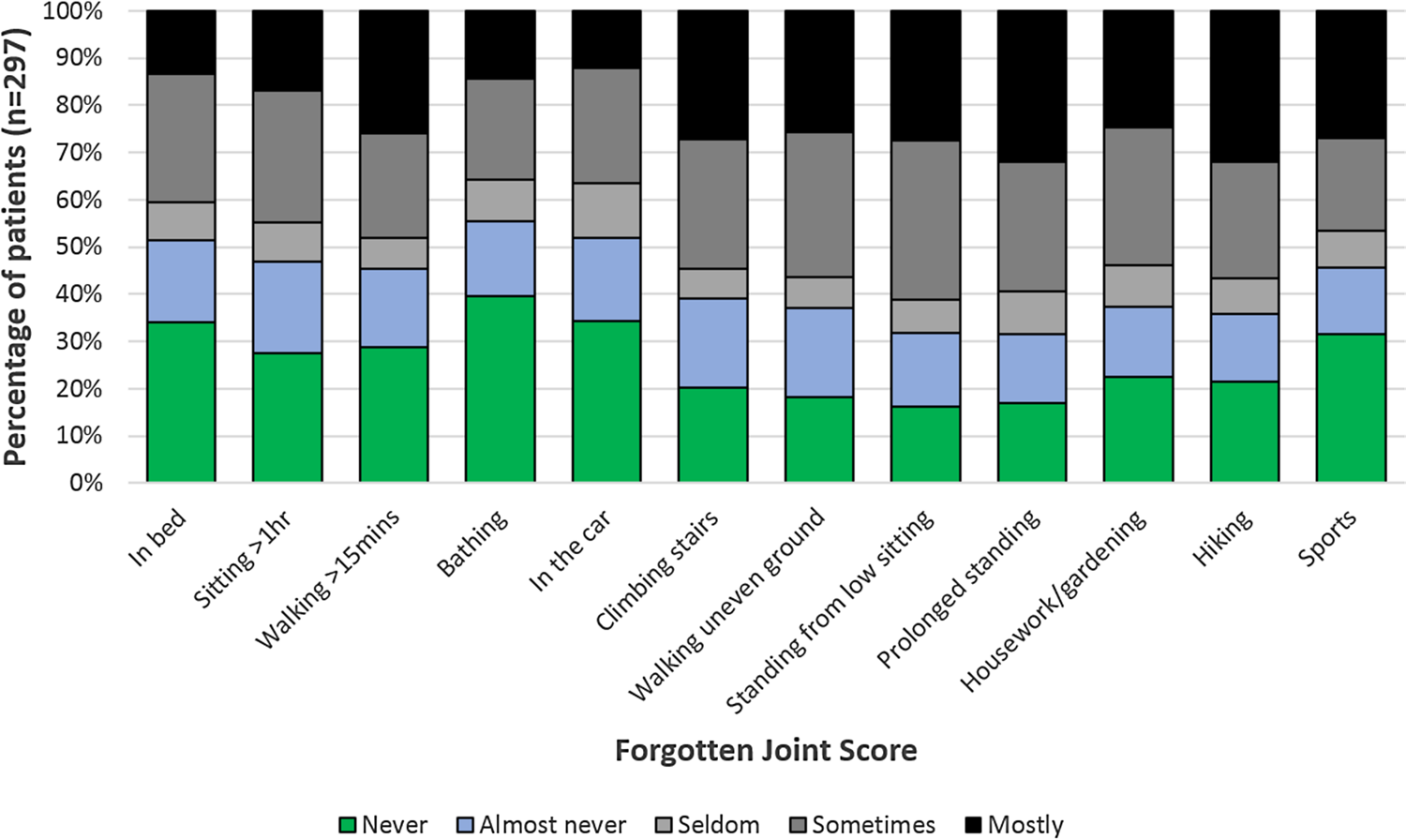
The Forgotten Joint Score (FJS) at 10 years

Oxford Knee Scores changed significantly over the study period (*p* < 0.001, Table 5; Fig. 3). The greatest improvement in mean population scores was in the first 6 months: 18.8 ± 6.9 to 34.3 ± 10.1 (*p* < 0.001). A statistically significant decline occurred between 5 and 10 years [37.3 ± 10.3 to 34.7 ± 10.5 (*p* < 0.001)] though this was less than the MCID. Mean improvement in OKS for individuals was 17.2 ± 9.7 at 1 year (*p* < 0.001) and 15.7 ± 11.5 at 10 years. The mean Forgotten Joint Score (FJS) was 48.2 ± 33.7 at 10 years (Fig. 4).

Patient satisfaction was 88.3% at 1 year, 88.0% at 5 years and 88.4% at 10 years. Of 35 patients dissatisfied at 5 years, 7 became satisfied by 10 years. Similarly, 9 who were satisfied at 5 years, were dissatisfied at 10. OKSs were worse in those dissatisfied at 10 years [20.3 ± 8.6 versus 35.2 ± 9.8 (*p* < 0.001)]. Nineteen patients (19 TKAs, 4%) reported anterior knee pain at clinical follow-up, one persistent after secondary resurfacing.

## Discussion

The key findings of this study are the high patient satisfaction (88%) and well maintained improvements in functional outcome scores at 10 years following a single-radius cruciate-retaining TKA with all cause survival of 97.9% (95% CI 96.5–99.3) and a worst-case analysis of 94.4% (92.1–96.8). This level of satisfaction compares well with the existing literature where satisfaction rates of 80–85% are consistently reported [3, 36]. The survival analysis is consistent with that reported by the National Joint Registry of England and Wales where a 10-year survival of 96.8% (96.3–97.2) is reported for 61,067 cemented cruciate retaining Triathlon TKAs, equivalent to eighth among 28 brands of TKA. Though joint registries report absolute implant survival with large sample sizes, this is not the only metric of implant success, or failure, and cohort studies offer beneficial complementary analysis including data on reoperations, modes of failure, radiographic loosening and PROMs. To date this is the only independent 10-year study of this popular implant of which > 2 million have been implanted worldwide to date. Very few patients were lost to follow-up in this study and long-term PROMs were obtained for 91% of those patients alive with intact TKAs at 10 years.

The mean individual improvement in OKS found in this study of 17.2 ± 9.7 by 1 year is favourable compared to the National PROMs database for England and Wales where a mean improvement in OKS 16.52 points at 6 months in 40,841 patients with 56% data capture is reported following primary TKA. Studies of longitudinal PROMs following TKA are scarce. Williams et al. [39] reported OKSs in 1547 TKA patients over 10 years demonstrating a mean pre-operative OKS of 19.5 (95% CI 18.8–20.2) in a population with similar demographics improving to 34.3 (33.5–35.1) at 1 year, peaking at 2 years and then slowly declining to 30.1 (29.1–31.1) at 10 years. They present population means at each time-point, rather than the improvements experienced by individual patients. Both population and individual OKS improvements here compare favourably with these scores. To limit any potential ceiling effects in high-functioning individuals, and to investigate the performance of this TKA in returning to specific functions, the Forgotten Joint Score was added to the 10-year follow-up questionnaire. This identified that patients were most aware of their arthroplasty during hiking, walking on uneven ground, when standing for a prolonged time, when standing from a low sitting position and when climbing stairs.

Satisfaction remained high throughout follow-up with 88% satisfied or very satisfied at every timepoint. This is favourable when compared to satisfaction rates of 81% consistently reported in the literature [3, 5, 36]. Changes in satisfaction over time have rarely been examined. Nilsdotter et al. [32] studied 102 patients over 5 years finding satisfaction to be unchanged from 1 to 5 years. Though overall rates remained the same in our study, 17/291 (5.8%) patients changed satisfaction status from 1 to 5 years, and 16/297 (5.4%) changed from 5 to 10 years. This phenomenon has also been reported by Clement et al. [7] who identified three groups of dissatisfied patients following TKA: those with early dissatisfaction at 1 year only; those with persistent dissatisfaction at 5 years; and those with late dissatisfaction only. All dissatisfied patients at 10 years in our study reported pain, usually diffusely. Pain has frequently been identified as an important predictor of dissatisfaction [3, 36].

The single-radius concept has performed well in joint registry data since it was introduced in the Scorpio TKA in 1996 with a 10-year risk of revision of 3.9% (95% CI 3.7–4.3). Though the current study lacks a multi-radius control group, single and multi-radius TKAs have been compared previously. Randomised control trials comparing the Triathlon single-radius TKA with multi-radius TKAs have found in favour of the single-radius design [9, 20, 22]. Hamilton et al. [22] reported significantly greater range of motion, more rapid recovery to 116% of quadriceps function compared to the contralateral side, greater satisfaction and reduced ‘worst daily pain’ following Triathlon (*n* = 104) compared to a multi-radius TKA (*n* = 108) over the 3 years of follow-up [22]. Collados-Maestre et al. [9] found significantly better knee society scores, WOMAC pain scores, range of motion and quadriceps strength in the single-radius group when comparing 118 single-radius and 119 multi-radius TKA designs at 5–7 years [9]. Biomechanical studies comparing single and multi-radius TKAs have found larger amounts of axial rotation in single-radius knees using dynamic fluoroscopy [19] and better power absorption during weight acceptance in single-radius TKAs at gait analysis [19]. These studies appear to support the patellofemoral friendly nature of single-radius designs. In our study, primary patella resurfacing was performed in 24 patients (5.2%), a rate markedly less than the 52% at reported for Triathlon TKAs in the NJR [2]. Four additional patients underwent secondary resurfacing for persistent anterior knee pain, one of whom continued to report anterior knee pain at 10 years. Anterior knee pain was reported clinically by 4% of patients. This is favourable compared to rates of 19–40% reported in the literature for patients following TKA [4, 38]. With no control group it is impossible to comment on whether the theoretically favourable patellofemoral kinematics of this implant with a longer quadriceps moment arm translate into clinical benefit. The most common reason for reoperation was knee stiffness which required MUA in ten cases. This incidence is consistent with previous reports of stiffness following TKA of 1–5% [30].

Non-randomised studies comparing outcomes of the Triathlon TKA to other multi-radius designs including the Duracon [10, 33] and the Kinemax Plus [14] have found in favour of the single-radius design in terms of pain relief [10, 14, 33], stability [10], degree of flexion [10, 33], walking and stair climbing ability [10], Knee Society Scores [33] and knee-related quality of life [14]. Favourable Knee Society Scores have been reported by Harwin et al. [23] in 2035 Triathlon TKAs in 1688 patients with 48-point improvements for pain and 22 for function at a mean of 21 months. Other non-randomised studies have found no differences in PROMs between single- and multi-radius posterior-stabilised TKAs [24].

The limitations of this study include a lower preoperative OKS response rate than at other assessment timepoints. As with all studies utilising PROM questionnaires, not all patients answer all the questions which can lead to responder bias. Attempts have been made to restrict any bias by including and reporting all available data in the analysis. Range of motion data were not collected and radiographs were not available for all patients. Radiographs were not all taken at the same timepoint reflecting the reality of clinical practice, though the most recent was used for analysis. Limitations are mitigated by only 8/462 (1.7%) TKAs being lost- to follow-up with validated PROMs on 93% of surviving patients at this timepoint and presentation of a worst-case-scenario analysis.

## Conclusion

This is the first independent study of 10-year survivorship and patient-reported outcome following cruciate-retaining single-radius TKA. It provides the first non-registry evidence for a widely used implant. It confirms that the good joint registry survival is coupled with excellent radiographic and functional outcomes and high patient satisfaction. The single-radius design theory utilised in this implant translates to a durable TKA prosthesis with favourable functional outcome and excellent patient satisfaction of 88%.
